# Medical students’ perception of dyad practice

**DOI:** 10.1007/s40037-014-0138-8

**Published:** 2014-07-30

**Authors:** Martin G. Tolsgaard, Maria B. Rasmussen, Sebastian Bjørck, Amandus Gustafsson, Charlotte V. Ringsted

**Affiliations:** 1 The Juliane Marie Centre, Copenhagen University Hospital Rigshospitalet, Blegdamsvej 9, 2100-O Copenhagen, Denmark; 2 Centre for Clinical Education, Capital Region of Denmark and Copenhagen University Hospital Rigshospitalet, Blegdamsvej 9, 2100-O Copenhagen, Denmark; 3 Department of Orthopaedic Surgery, University Hospital Slagelse, Slagelse, Denmark; 4 Department of Orthopaedic Surgery, University Hospital Hvidovre, Hvidovre, Denmark; 5 The Wilson Centre and Department of Anesthesia, University of Toronto and University Health Network, Toronto, Canada

**Keywords:** Dyad practice, Peer-learning, Clinical skills training

## Abstract

Training in pairs (dyad practice) has been shown to improve efficiency of clinical skills training compared with single practice but little is known about students’ perception of dyad practice. The aim of this study was to explore the reactions and attitudes of medical students who were instructed to work in pairs during clinical skills training. A follow-up pilot survey consisting of four open-ended questions was administered to 24 fourth-year medical students, who completed four hours of dyad practice in managing patient encounters. The responses were analyzed using thematic analysis. The students felt dyad practice improved their self-efficacy through social interaction with peers, provided useful insight through observation, and contributed with shared memory of what to do, when they forgot essential steps of the physical examination of the patient. However, some students were concerned about decreased hands-on practice and many students preferred to continue practising alone after completing the initial training. Dyad practice is well received by students during initial skills training and is associated with several benefits to learning through peer observation, feedback and cognitive support. Whether dyad training is suited for more advanced learners is a subject for future research.

## Introduction

The increasing numbers of medical students internationally is a growing concern from a training perspective. These concerns have led to the search for efficient and effective educational strategies that address issues of quality of training, cost, and clinical teacher resources. This may include the use of testing technologies, structured supervision and feedback or strategies to promote self-regulated learning, self-direction and cooperative learning. In an experimental study by Tolsgaard et al., students who trained patient management skills in pairs (dyads) demonstrated significantly higher learning outcomes than students who trained alone (singles) [1]. The students in the dyad group were also significantly more confident after completed training compared with the students who practised alone. Recent studies on simulation-based training confirm these findings by demonstrating non-inferiority of dyad practice compared with single practice in a group of residents [2, 3]. These results are remarkable as the dyads received only half the hands-on training time of the singles. Previous research from the field of psychology has suggested that reduced cognitive load, improved confidence, and positive effects of observation are responsible for the beneficial effects of dyad training [4–6]. However, there is currently little knowledge of the proposed mechanisms of action and few studies have addressed how medical students perceive working in pairs. The aim of this pilot study was therefore to explore the reactions and attitudes of medical students, who were instructed to work in pairs during clinical skills training.

## Methods

This pilot study included follow-up survey data from the intervention group of a previously published randomized controlled trial; the intervention has been described in full detail elsewhere [1]. Twenty-four fourth-year medical students without prior clinical experience underwent a four-hour course on how to manage patient encounters. The primary focus of this course was on patient history taking and on how to perform a standard physical examination. The students then practised for four hours on different case scenarios using standardized patients. The students were instructed to practice together and take turns as the active participant. Discussion was allowed but there were no explicit instructions on how the students should interact during practice. Two weeks after completed training, the students were surveyed on how they perceived training in pairs in terms of advantages and disadvantages of this type of training. The survey items included four open-ended questions: 1) What do you think about training in pairs? 2) Which benefits for learning did dyad training provide? 3) Which limitations to learning does dyad training provide? 4) Describe how you interacted with your training partner.

The survey responses were analyzed by two of the authors (MGT and CR) and categorized according to emerging themes without the use of coding software. Agreement on themes was reached through discussion until consensus. The authors used a constructivist approach to analyze data. The theoretical lenses used by the authors during the data interpretation were shaped by existing dyad literature from the field of psychology [3–6]. Ethical approval was obtained from the Ethical Committee for the Capital Region, Denmark, prior to undertaking this study.

## Results

The response rate was 100 % (24 respondents). The students were generally positive about training in pairs. Responses and themes are shown in Table 1. The students felt dyad practice improved their self-efficacy through the social interaction with peers, provided useful insight through observation, and contributed with shared memory of what to do, when they forgot essential steps of the physical examination of the patient: *‘By observing, you free the capacity to think about what to do better’ [and] ‘it was an advantage to get feedback from each other after each encounter* – *my partner helped me point out the things I forgot.’* The students in the present study indicated that a significant part of the benefit of dyad practice was due to peer feedback and improved confidence*: ‘Having a partner makes you less nervous and more confident during the first patient encounters.’* Yet some students indicated that the social dynamics within the dyad could be harmful to learning due to the psychological pressure of being observed by a peer. The most common concern about dyad practice was the reduced amount of hands-on time: *‘Less hands*-*on time worries me!’ [and] ‘it decreases the amount of direct contact with patients’.* While appreciating dyad practice in the initial stages of learning, students expressed concerns about this training format in later stages of learning: *‘It was nice to have a partner during the first few patient encounters but that was enough. To become experienced I would have to practice alone at some point’.*

**Table 1:** Thematic analysis of survey results

Themes	Student responses
(1) *What do you think about training in pairs of two?*	
Initial versus further training	‘I would not like to be two every time—only in the beginning’
	‘Reasonable to have someone as a sparring partner the first times’
	‘Helped breaking the ice’
	‘Good way to break the first consultations—a mix would be perfect’
	‘Important that you try both being alone and together’
	‘Good idea the first few times’
Social dynamics	‘Learning outcome depends on the chemistry and levels between partners…’
Observation	‘Observing another student managing the patient encounter was very helpful’
	‘Good to observe during patient encounter and to talk about it afterwards’
	‘You see what works and what seems awkward’
	‘Possibility of observation under peaceful conditions’
	‘It was difficult to interrupt and then it really does not matter’
	‘It was lovely to be able to have someone observe you while being able to have someone to discuss the case with later on’
	‘Exciting to watch fellow students and really good to have opportunity to be reminded of what you don’t remember yourself’
	‘Helpful to have someone to observe and someone to observe you’
	‘You learn something from seeing the other managing the examination—that way you figure things out in the situation’
	‘In the situation my performance becomes weaker because I become stressed by someone else observing me. This is only the first time—you become better in the following encounters because you have learned from your partner’
	‘Helpful to observe another person perform the examination’
	‘Helpful to learning to observe another person’
Confidence	‘It felt safe to be two to begin with’
	‘It was nice and comforting not to be alone’
	‘It made the first few encounters less awkward ‘
	‘Provided some confidence’
	‘You feel less insecure in the beginning’
	‘Dyad training made it more safe in the beginning’
	‘Cool to have this kind of back-up’
Feedback	‘It was nice to have feedback immediately after finishing the encounter’
	‘Good to provide each other with missing information’
	‘It was good to discuss findings and missing information in pairs after each encounter’
	‘Nice to support each other with the things you forget’
	‘Possibility of supplementing each other’
	‘Good to have someone to discuss with during and after the encounter’
	‘Helpful to have someone to remember the things you forgot’
	‘Feedback is an advantage, although your partner is just as new as you are’
	‘Comforting and interesting to get advice from a peer’
Learning	‘You learn better when you had someone to talk to’
(2) *Which benefits for learning did dyad training provide?*	
Confidence/social security	‘Support each other’
	‘Less insecurity and just pleasant’
	‘Safeness in the learning process’
	‘Potential insecurity is removed when working in pairs’
	‘It removes some of the first-time insecurity when you have someone with you carrying a check-list’
	‘Beneficial social effects—everything is more manageable when you are not alone the first time’
	‘No insecurity, if that should be a problem’
	‘Safety’
Reflection	‘The option to discuss patients and […] to evaluate diagnoses’
	‘If you are ‘on’ it is difficult to think about what you are doing’
	‘You learn things faster as you become more aware of your own mistakes and of things you forgot to ask’
	‘By observing you free capacity to think about what to do better’
	‘Allows opportunity for more in depth history taking and physical examination’
	‘Able to supplement each other and discuss diagnoses and complications’
	‘You learn much from each other’s mistakes’
	‘I get inspired with new ideas about how and what questions to ask’
Feedback	‘Evaluating own work and supplying suggestions for improvement’
	‘Less need for asking questions to the supervising officer’
	‘Good way to give feedback to each other’
	‘The one who observed pointed out the things you forgot’
	‘An advantage to evaluate and being observed’
	‘Feedback right away’
Learning	‘Better insight, easier to remember, better learning’
(3) *Which limitations to learning does dyad training provide?*	
Independence/individualism	‘I get impatient and I want to examine myself!’
Social dynamics	‘It is irritating to work with someone, who you are not on the same page with’
	‘You aren’t under the same state of pressure’
	‘One can feel shy when another person observes you’
	‘You can feel monitored by your peer’
	‘If your partner is very much better than you, you do not benefit that much from it’
Hands-on	‘It is difficult to interrupt and you only get half the training time’
	‘Less active yourself’
	‘Less experience in the physical examination’
	‘Fewer cases’
	‘You see fewer patients’
Quality of encounter	‘You don’t recognize your own weaknesses because there’s someone to support you’
	‘Decreases interaction with patients—not significantly though’
	‘Risk of being less thorough because you always have someone to remind you of things you forget’
	‘Sometimes you may be less focused when you are not the one who is active’
(4) *Describe how you interacted with your training partner?*	
Case-based discussion	‘Discussion of our encounter and each other’s deficits…discussion of symptoms, work-up and treatments options’
	‘Exchanging ideas’
	‘Good feedback after encounters’
	‘Got advice from each other—supply each other with additional information’
	‘Discussion of what you have done and what you forgot—was very useful’
	‘We talked freely about differential diagnoses and patient work-up’
	‘Discussing diagnoses and being a spare memory’
	‘Discussing the case at hand’
	‘Discussion of the case and of the history taking and physical examination’
	‘Supplying each other with questions we forgot to ask’
	‘Discussion of treatment options—adding up information’
	‘Interaction and creation of common write-up and diagnoses’
	‘Making sure that you got all the important information’
	‘You can become blinded by all the possibilities but having a partner makes you see the obvious’
	‘The one, who was the ‘doctor’ asked after each element in the history taking if he/she forgot something’
	‘Discussing and agreeing before writing the journal’
	‘Discuss the encounter’
	‘Cooperate about a joint treatment plan and suggestions for diagnoses’
	‘Discussion and evaluation of performance’
	‘Discussion of what to do differently next time’
Social support	‘Support from buddy if unconfident’
Deliberate practice	‘We had a relevant discussion of why we did as we did and what to improve’

The students reported that the interaction with a peer provided them with a structured approach to case-based learning: ‘*We used each other as checklists’* *and ‘we used each other for systematic case discussion’*. Finally, the students reported that dyad practice stimulated deliberate practice strategies: *‘We had a relevant discussion of why we did as we did and what to improve’* and *‘we talked about what we remembered and what we forgot and what to do better the next time’.*

## Discussion

This study aimed to explore students’ reactions to dyad practice and to probe students regarding both positive and negative aspects of this training format. Our results confirm the theoretical underpinnings of dyad practice with regard to shared memory, improved self-efficacy, and positive effects mediated through observation and peer feedback [7]. Although experimental studies conducted within the field of psychology and medical education provide evidence of the effectiveness of dyad practice, this study stresses the need to further explore, which learners may and may not benefit from dyad practice and under what conditions. Research from the field of psychology suggests that collaboration may reduce the risk of cognitive overload due to united memory and shared information processing [8]. These aspects relate well to the results of our study, demonstrating that students perceived considerable social and psychological benefits from dyad training. However, in contrast to the motor skills learning literature suggesting that observation is the most significant factor responsible for learning during dyad practice [9], the students in the present study indicated that part of the benefit stems from feedback and shared memory with the other peer. While previous studies on dyad practice have focused on motor skills learning, the task being trained in the present study involved knowledge, technical skills, and diagnostic reasoning skills. The discrepancies between our findings and the existing literature may therefore reflect the different tasks being practised, as motor skills learning may rely more on observation than tasks that depend on problem-solving and diagnostic reasoning skills.

Although the students reported several benefits from practising in pairs during initial practice, they also expressed concerns regarding dyad practice during later stages of learning. By contrast, Crook & Beier suggested that dyad training may be harmful in early acquisition of declarative knowledge [10] and Shanks et al. recently hypothesized that the benefits from training in pairs stems from using advanced learners, who have the experience needed to provide meaningful feedback to each other during dyad practice [2]. However, more advanced learners may not appreciate working in pairs, as the students in our study called for increasing amounts of individual hands-on time and less time for observation after only four hours of practice. Hence, students may wish to practice alone once the advantages from working in pairs in terms of increased confidence and shared memory no longer outweigh the limitations of reduced hands-on practice.

The high response rate was a major strength of this study. The students who participated in this study were highly motivated volunteers, which may explain the fact that all of them contributed with written evaluations. Furthermore, it is customary in our institution that all courses are followed by written evaluations so this may also explain the high response rate. There are several limitations to this study. The largest one is that we used a survey rather than interviews or observations, which would have enabled a more in-depth analysis of learning mechanisms during dyad practice. However, this was a pilot study that aimed to explore students’ perceptions of dyad practice, and the purpose was to generate questions and to probe for reactions rather than to provide an exhaustive explanatory framework for dyad practice. Future research is needed to explore how the quality of peer feedback may affect learning during dyad practice and under which conditions dyad practice is most effective.

## Conclusion

Dyad practice is well received by students during initial skills training and is associated with several benefits to learning through peer observation, feedback and cognitive support. Hence, dyad practice is supported by both empirical evidence as well as learning theories and should be considered for future basic clinical skills training. Whether dyad training is suited for more advanced learners is a subject for future research.
